# Whole exome sequencing in Finnish families identifies new candidate genes for osteoarthritis

**DOI:** 10.1371/journal.pone.0203313

**Published:** 2018-08-29

**Authors:** Sini Skarp, Olli-Pekka Kämäräinen, Gong-Hong Wei, Eveliina Jakkula, Ilkka Kiviranta, Heikki Kröger, Juha Auvinen, Petri Lehenkari, Leena Ala-Kokko, Minna Männikkö

**Affiliations:** 1 Center for Life Course Health Research, Faculty of Medicine, University of Oulu, Oulu, Finland; 2 Faculty of Biochemistry and Molecular Medicine, University of Oulu, Oulu, Finland; 3 Department of Orthopaedics and Traumatology, University of Helsinki and Helsinki University Hospital, Helsinki, Finland; 4 Department of Orthopaedics and Traumatology, Jyväskylä Central Hospital, Jyväskylä, Finland; 5 Department of Orthopaedics and Traumatology, Kuopio University Hospital and Kuopio Musculoskeletal Research Unit, University of Eastern Finland, Kuopio, Finland; 6 Department of Anatomy and Cell biology and Surgery Clinic, Medical Research Center, University of Oulu and Oulu University Hospital, Oulu, Finland; 7 Connective Tissue Gene Tests, Allentown, PA, United States of America; 8 Northern Finland Birth Cohort, Faculty of Medicine, University of Oulu, Oulu, Finland; German Cancer Research Center (DKFZ), GERMANY

## Abstract

**Introduction:**

Osteoarthritis (OA) is the most common degenerative joint disease and one of the major causes of disability worldwide. It is a multifactorial disorder with a significant genetic component. The heritability of OA has been estimated to be 60% for hip OA and 39% for knee OA. Genetic factors behind OA are still largely unknown. Studying families with strong history of OA, facilitates examining the co-segregation of genetic variation and OA. The aim of this study was to identify new, rare genetic factors and novel candidate genes for OA.

**Methods:**

Eight patients from three Finnish families with hip and knee OA were studied using whole exome sequencing. We focused on rare exonic variants with predicted pathogenicity and variants located in active promoter or strong enhancer regions. Expression of identified candidate genes were studied in bone and cartilage tissues and the observed variants were investigated using bioinformatic analyses.

**Results:**

Two rare variants co-segregated with OA in two families. In Family 8 a missense variant (c.628C>G, p.Arg210Gly) was observed in the *OLIG3* gene that encodes a transcription factor known to be associated with rheumatoid arthritis and inflammatory polyarthritis. The Arg210Gly variant was estimated to be pathogenic by Polyphen-2 and Mutation taster and the locus is conserved among mammals. In Family 12 the observed variant (c.-127G>T) was located in the transcription start site of the *FIP1L1* gene. FIP1L1 participates in the regulation of polyadenylation. The c.-127G>T is located in the transcription start site and may alter the DNA-binding of transcription factors. Both, OLIG3 and FIP1L1 were observed in human bone and cartilage.

**Conclusion:**

The identified variants revealed novel candidate genes for OA. *OLIG3* and *FIP1L1* have specific roles in transcription and may effect expression of other genes. Identified variants in these genes may thus have a role in the regulatory events leading to OA.

## Background

Osteoarthritis (OA) is the most common degenerative joint disease and one of the leading causes of disability in the world [1]. The incidence of OA is increasing as the population ages and OA has been predicted to become the greatest single cause of disability by 2030 [2].

OA is characterized by progressive degradation of the joint cartilage. It includes synovial inflammation and bone remodeling causing pain, and eventually leading to loss of joint function. Most often it affects the joints in the knee, hip, or hand, but can affect any joint. OA is a complex disease that has a strong genetic background with heritability estimations of 39% and 65% for hand and knee OA, respectively [3,4] and 60% for hip OA [5].

Genetic factors behind OA have been studied in both family and case-control datasets using linkage analyses and candidate gene approaches, as well as in genome-wide association studies (GWAS) carried out in large population cohorts. Although, several genetic loci have been associated with OA the underlying genetic variants and genes remain elusive. Based on these numerous studies the genetic background of OA is likely to be affected by common (minor allele frequency, MAF > 0.05), low frequency (0.01 < MAF < 0.05) and rare variants (MAF < 0.01) [6,7]. Large GWASs have usually focused on the common genetic variation, although study of Islandic population identified variants with rare or low frequency in n cartilage oligomeric matrix protein (*COMP*) gene and chondroadherin like (*CHADL*) gene [8]. The variant in *COMP* was specific to Islandic population [8]. Families with multiple early onset OA cases in several generations are of particular interest as there might be a stronger genetic effect. For example, candidate gene studies of families with an early onset of OA have previously identified variants in the genes encoding for collagens II and XI [9,10]. Studies of families with OA have identified rare or low frequency variants in loci 1p31 and 2q33.3 [11,12]. In general, selective pressure keeps pathogenic variants from reaching a high frequency in human populations and thus rare coding variants are typically more harmful and population specific compared to common variants [13,14].

The Finnish population is particularly advantageous for studying familial cases of common diseases due to its population history [15]. It has undergone multiple historic bottlenecks followed by population expansion [16]. Due to the resulting founder effect, functional and low-frequency variants have become enriched in the Finnish population [17,18].

The objective of this study was to identify rare variants co-segregating with OA in Finnish families.

## Methods

### Families

**Fig 1:**
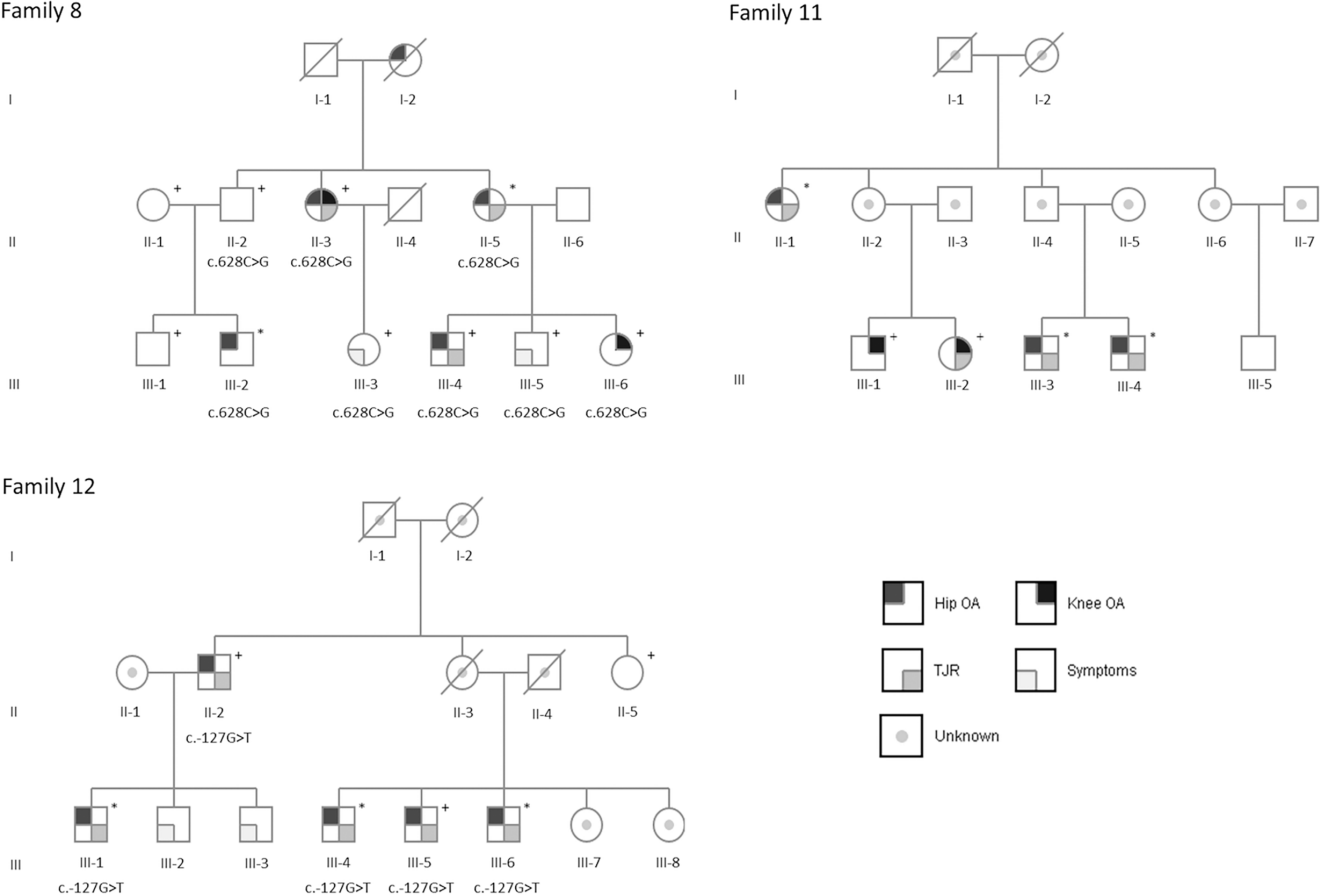
Pedigrees of Families 8 and 12. The patients included in whole exome sequencing are marked with an asterisk and individuals included in genotyping are marked with a plus symbol. Deceased are marked with diagonal line through symbol. In Family 8 the carriers of the *OLIG3* variant c.628C>G, p.Arg210Gly are marked with c.628C>G. In Family 12 the observed variant c.-127G>T is located on the *FIP1L1* gene 5’UTR. Abbreviations: Hip OA = Osteoarthritis of the hip, Knee OA = Osteoarthritis of the knee, TJR = Total joint replacement surgery, Symptoms = Individual has no OA diagnosis, but experiences pain and/or joint stiffness.

Three families diagnosed with an early onset hip and knee OA were studied using whole exome sequencing. These families were part of a previously described data set of 15 families [19] and were recruited during the years 1998–2000 and 2003–2006. For the whole exome sequencing we chose families with at least two affected family members in more than one generation (Families 8, 11 and 12, Fig 1). Exome sequencing was performed for three patients from Families 11 and 12, and two from Family 8. They had been diagnosed with OA of the hip under the age of 55, and seven of them had undergone total joint replacement surgery (TJR) due to OA. Characteristics of the families are presented in Table 1. None of the individuals had any other inflammatory joint disease such as rheumatoid arthritis (RA). There was also no evidence of a skeletal or chondrodysplasia.

**Table 1:** Characteristics of the families.

Fam	N (M/F)	Osteoarthritis			Healthy	Symptoms	Unknown	MAge	MAgeDiag
		Hip	Knee	Hip&Knee				(range)	(range)
8	14 (8/6)	4	1	1	6	2	0	44.25 (30–70)	38.3 (30–55)
11	14 (8/6)	3	2	0	1	0	8	66.0 (58–89)	40.0 (30–50)
12	15 (9/4)	5	0	0	1	2	7	64.25 (47–78)	51.0 (50–52)

Abbreviations: Fam = Family, N = Number of individuals, M = Male, F = Female, MAge = Mean age, MAgeDiag = Mean age at the time of diagnosis. Mean age is calculated using information of all individuals in the family and Mean age at the time of diagnosis is calculated from the OA affected individuals.

In addition to the samples subjected to exome sequencing, DNA (deoxyribonucleic acid) samples were available from seven other family members from Family 8 (3 unaffected, 4 affected), two family members from Family 11 (both affected) and three family members from Family 12 (2 affected, 1 unaffected).

This study was approved by the Ethics Committee of the Northern Ostrobothnia Hospital District and family members as well as the donor of the osteochondral specimen gave their informed written consent.

### Whole exome sequencing

Genomic DNA was extracted from 3 ml of whole blood treated with ethylenediaminetetraacetic acid (EDTA) using standard protocols. The exome sequencing data was acquired through a commercial service (BGI, Hong Kong), who provided the exome capture, alignment and variant calling. The SureSelect 51M Capture Kit (Agilent Technologies) was used for the exome capture. The targets were sequenced using the IlluminaHiSeq2000 100PE platform and the reads were aligned to the GRCh37/hg19 human reference genome. The Genome Analysis ToolKit (GATK) [20] was used for variant calling and filtering. Variants were filtered out according to quality parameters/threshold values, which for SNVs are the root mean square of the mapping quality (MQ) < 40, Haplotype score > 13 and genotype quality (GQ) < 20, and for indels are the quality by depth (QD) < 2.0, the Phred-scaled p-value using Fisher’s Exact Test (FisherStrand) > 200.0 and the u-based z-approximation from the Mann-Whitney Rank Sum Test for mapping qualities (MappingQualityRankSumTest) < -20.0.

### Analysis of whole exome sequencing data

Variants shared by the affected family members were extracted and variants, which were found in the unaffected individuals or in the in-house exome set (N = 71) or had a MAF > 1% in the 1000Genomes database were filtered out.

Functional annotation was performed for the remaining rare variants using database for nonsynonymous SNPs' functional predictions (dbNSFP) [21] through ANNOVAR [22] (version Aug 2013) to identify variants with estimated pathogenicity (SIFT, PolyPhen-2, MutationTaster) and variants affecting active promoters or strong enhancer regions (the Encyclopedia of DNA Elements [ENCODE] databases for chromHMM estimations for HSMM and GM12878 cells). Thresholds stated in the dbNSFP and ANNOVAR guidelines were used to determine pathogenic estimation: SIFT≤0.05, Polyphen-2 ≥ 0.957 or MutationTaster ≥ 0.85. All annotations were done according to the ANNOVAR manual. Rare variants without any predictions were not excluded at this stage, however. The Sequencing Initiative Suomi (SISu) database (N = 6118) representing the Finnish population (http://www.sisuproject.fi/), the Exome Aggregation Consortium (ExAC) (http://exac.broadinstitute.org) and the Genome Aggregation Database (gnomAD) (http://gnomad.broadinstitute.org/) were used as references.

All variants with estimated pathogenicity and those affecting active promoters or strong enhancer regions were validated using capillary sequencing (ABI3500xL Genetic Analyzer, Applied Biosystems). DNA samples from additional family members were genotyped using the same method to study the co-segregation of variants in families.

### Bioinformatic analyses

RegulomeDB [23] and Enhancer Element Locator (EEL) software [24] were used to predict whether the c.-127G>T variant in the *FIP1L1* (Factor Interacting With PAPOLA And CPSF1) 5’ untranslated region (5’UTR) directly affects DNA-binding motifs of certain transcription factors (TF). The conservation of the Oligodendrocyte transcription factor 3 (OLIG3) protein sequence at the p.Arg2010Gly site was visualized using Constraint-based Multiple Alignment Tool (COBALT) [25].

### Osteochondral specimen

Bone and cartilage tissues are not included in the public databases such as Genotype-Tissue Expression portal (GTEx, www.gtexportal.org) and Human protein atlas (www.proteinatlas.org). To study the expression of the identified genes in bone and cartilage, tissue samples were collected from one unrelated patient who had undergone total knee replacement therapy at Oulu University Hospital in 2014. The Ethics Committee of the Northern Ostrobothnia Hospital District approved of the study protocol for tissue collection and gene analysis and the patient gave a written informed consent in accordance with the Declaration of Helsinki. Tissue samples were stored at -70°C before RNA extraction.

### Total ribonucleic acid (RNA) extraction from bone and cartilage tissue

Each tissue sample was cut into approximately 5 mm thick and 2 mm wide pieces and stored in an RNAlater solution (Ambion). Four pieces of each tissue sample were used for RNA extraction reaction. The samples were pulverized on a metal surface pre-cooled in liquid nitrogen and then homogenized in 4 M guanidinium isothiocyanate. A pellet was collected following cesium chloride (5.7 M) gradient centrifugation. The pellet was dissolved in 300 μl of diethyl pyrocarbonate treated water (DEPC-H2O) before adding 20 μl of proteinase K (20 mg/ml) and 500 μl of SET-buffer (1% sodium dodecyl sulfate (SDS), 4 mM of EDTA, 10 mM of tris(hydroxymethyl)aminomethane (Tris), and incubated with constant agitation for 1.5 hours in 37°C. Total RNA was extracted using 25:24:1 phenol:chloroform:isoamylalcohol. The samples were mixed and centrifuged at 20,000 rcf at room temperature for four minutes. The upper aqueous phase was collected and RNA was precipitated using 3 M sodium acetate (NaAc) and absolute ethanol. Samples were centrifuged for 15 min at + 4°C and dried using SpeedVac (Savant) before dissolving the RNA into 50 μl of DEPC-H2O.

### Messenger RNA (mRNA) expression in bone and cartilage

The quality of the RNA was determined with the QIAxcel Advanced system and RNA screening kit (Qiagen). Some level of degradation was observed. Complementary DNA (cDNA) was synthesized from the extracted total RNA using the iScript cDNA Synthesis Kit (Bio-Rad). A 327 base pair (bp) region of the *FIP1L1* cDNA and a 343 bp region of the *OLIG3* cDNA were amplified using polymerase chain reaction (PCR) and an AmpliTaq gold DNA polymerase enzyme (Applied Biosystems). These PCR products were used as a template in further amplification, where the product sizes were 109 bp for *FIP1L1* and 343 bp for *OLIG3*. The presence and size of the PCR product was determined by electrophoresis using the QIAxcel Advanced system and QIAxcel ScreenGel Software (Qiagen). Primer sequences are available on request.

## Results

### Whole exome sequencing

Variant calling resulted in an average of 78,638 SNVs and 8,922 short insertion/deletion variants (ins/del) per sample. The average sequencing depth in the target area was 67.06X. Filtering by quality resulted in an average of 71,199 SNVs and 8,846 ins/del variants per sample. All variants that were shared by the exome sequenced family members and not found in the in-house exome set (N = 71) are listed in S1 Table. Functional annotation identified 37 variants in Family 8, twelve variants in Family 11 and eleven variants in Family 12. After validation and genotyping in additional family members only one variant in Family 8 and one variant in Family 12 were found to be co-segregating with OA (Fig 1, Table 2). None of the variants co-segregated with OA in Family 11.

**Table 2:** Variants identified co-segregating with OA in whole exome analysis.

Fam	Gene	Chr:Locus	Variant	rsID	ExAC		gnomAD		Path. Est.
					Eur	Fin	Eur	Fin	
8	*OLIG3*	6:137814680	c.628C>G, p.Arg210Gly	rs750429448	4.4x10^-5^	0	1.2x10^-5^	0	SIFT:T, PP-2:P, MT: D
12	*FIP1L1*	4:54243878	c.-127G>T	rs550992103	NC	NC	0.003531	0.01317	SIFT:NA, PP-2:NA, MT: NA

Abbreviations: Fam = Family, ExAC Finn = Exome Aggregation Consortium ExAC Finnish population, Eur = Exome Aggregation Consortium European population, gnomAD = Genome Aggregation Database, NC = No coverage, PP-2 = PolyPhen-2, MT = MutationTaster, D = Damaging, P = Probably damaging, T = Tolerated, Path. Est. = Pathogenicity estimations

The observed variant in Family 8 was a missense variant c.628C>G, p.Arg210Gly (NM_175747) in the *OLIG3* gene. It was carried by all the family members with diagnosed OA or showing some symptoms of OA. This was observed as heterozygous in one individual in the ExAC and gnomAD datasets (Table 2). Variant was not available in the SISu database.

In Family 12 one variant, c.-127G>T, in the 5’UTR of *FIP1L1* gene was observed in five affected family members. This variant had frequency of 0.0035 in the gnomAD database (Table 2). Variant was not available in the SISu database.

### Bioinformatic analyses

**Fig 2:**
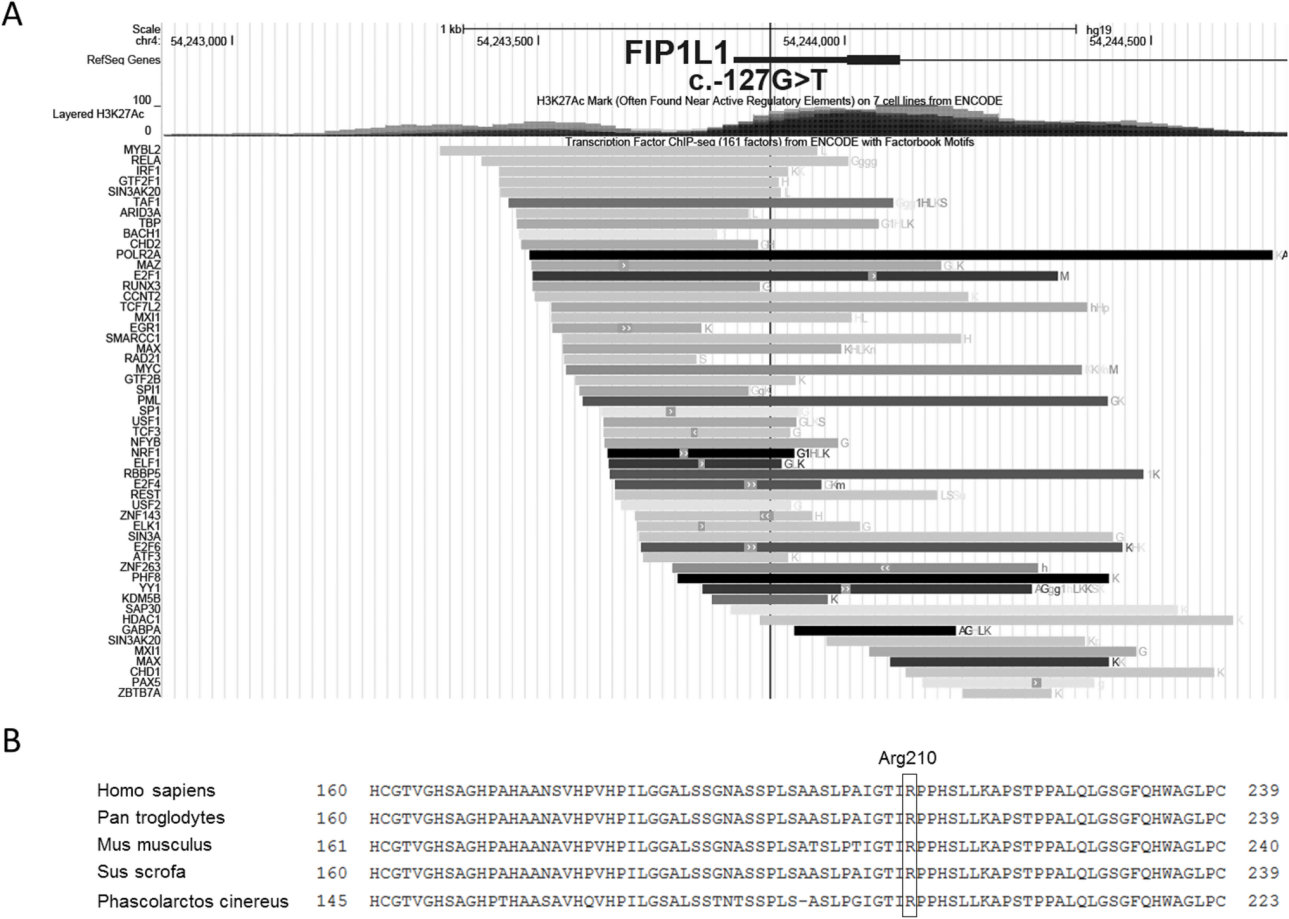
Variant loci. A) The c.-127G>T variant is located at the transcription start site of FIP1L1. The location of the variant is marked with a vertical bar. Transcription factor binding sites are shown with black and grey bars below the layered H3K27ac signals. The results were visualized with the UCSC genome browser utilizing ENCODE project data sets. B) Protein sequence alignment of OLIG3. The locus of p.Arg210Gly is highlighted. The 210^th^ arginine is conserved across mammals. Alignment shown from 160^th^ amino acid to 239^th^ amino acid. The alignment is drawn using Constraint-based Multiple Alignment Tool (COBALT).

**Fig 3:**
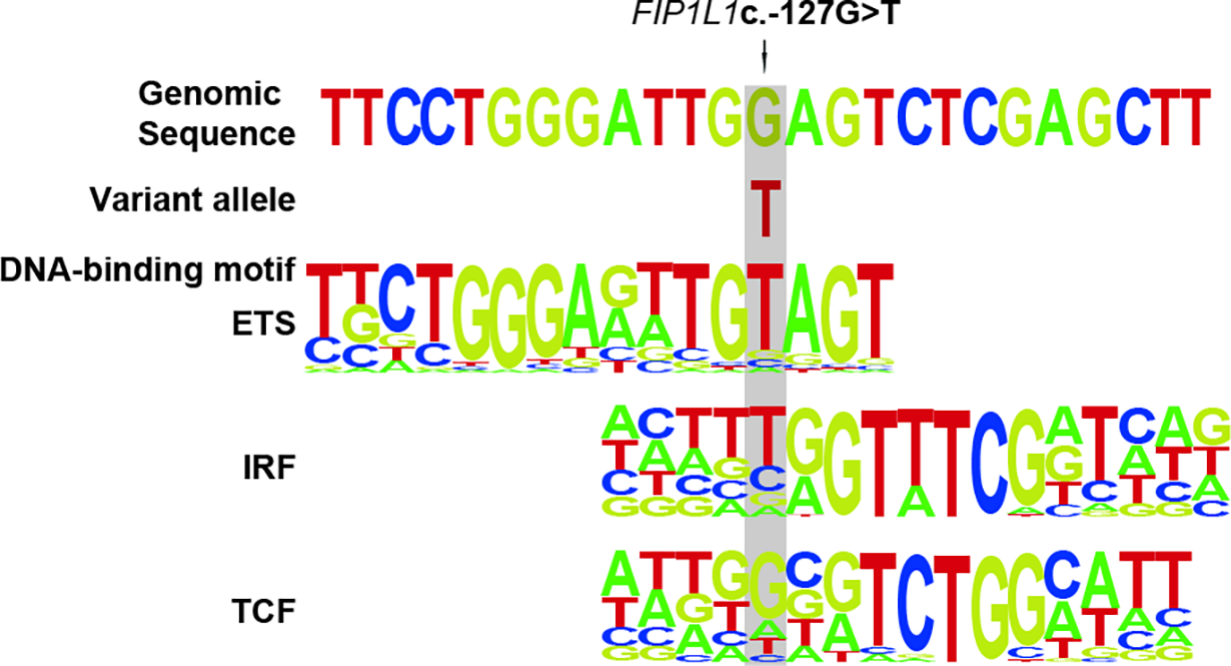
Alignment of genomic sequence surrounding the *FIP1L1* c.-127G>T variant and DNA-binding motifs for ETS, IRF and TCF transcription factors.

The area around *FIP1L1* c.-127G>T harbors high signals for acetylation lysine 27 histone 3 (H3K27ac) and several transcription factor binding sites (TFBS) (Fig 2) that are characteristic for active transcription starting sites (TSS). H3K27ac is an epigenetic marker for the activation of gene expression [23] and the binding of TFs at TSS is a hallmark of the start of transcription [24]. The prediction using RegulomeDB showed that the *FIP1L1* c.-127G>T variant is likely to affect TF binding, but the program was unable to match the exact TF DNA-binding motif. The EEL analysis of the region revealed that this variant may alter the DNA-binding of several transcription factors (Fig 3), including IRF1 (IRF Family), SPI1, ELF1, ELK1, GABPA (ETS Family) and TCF3 (TCF Family) that have ChIP-seq signals in this region, and thus their chromatin binding may be affected due to the observed variant.

The OLIG3 p.Arg210Gly variant was considered pathogenic by Polyphen-2 and Mutation taster, but benign by SIFT. The pathogenicity estimation by SIFT is based on conservation whereas the other two (Polyphen-2 and Mutation taster) consider also biochemical information available. The conservation of OLIG3 Arg210 locus is presented in Fig 2. The multiple alignment of OLIG3 protein sequence shows that Arg210 is conserved among mammals. However variation in the locus occurs in more distant vertebrata members.

### mRNA expression in bone and cartilage

**Fig 4:**
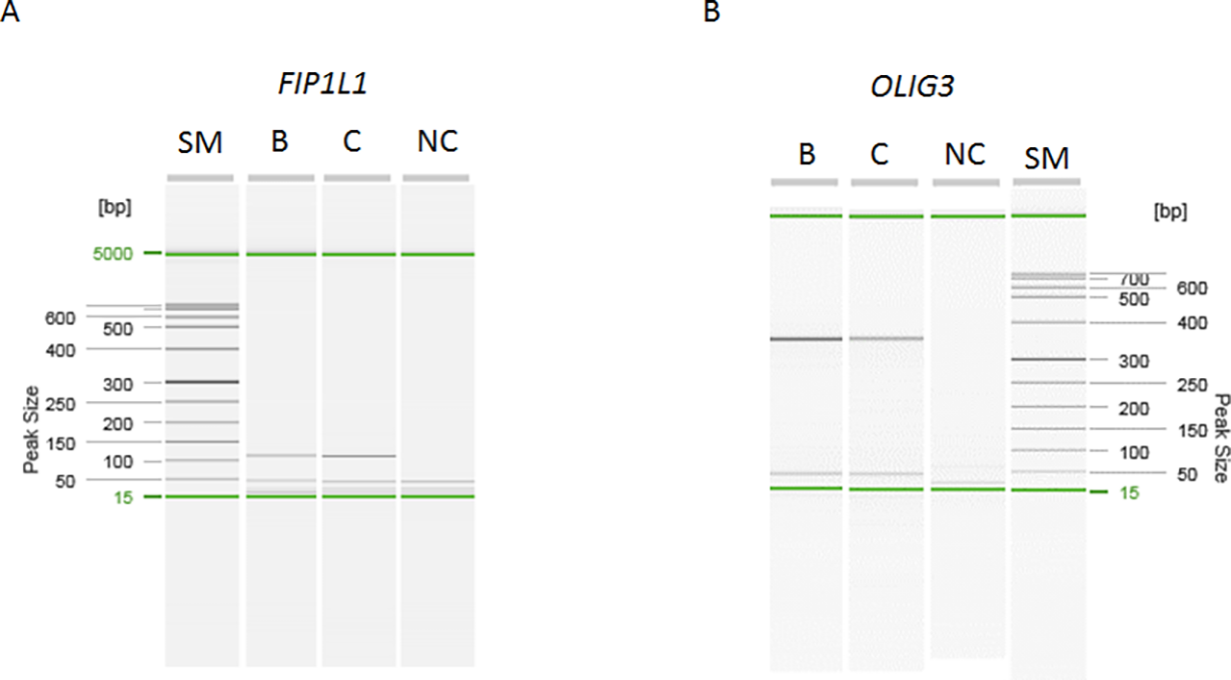
Expression of FIP1L1 and OLIG3 mRNA in bone and cartilage tissues. The size of the PCR fragments was determined by electrophoresis using the QIAxcel Advanced system and QIAxcel ScreenGel Software (Qiagen). The 5,000 bp and 15 bp size markers are visible as green fragments in all the samples. The size of the *FIP1L1* qPCR fragment was 109 bp and the size of *OLIG3* PCR fragment was 343 bp. Abbreviations: SM = Size Marker, B = Bone sample, C = cartilage sample, NC = negative control, bp = base pair.

The expression of *FIP1L1* and *OLIG3* messenger RNAs (mRNA) in human bone and cartilage tissues was observed using PCR. Products of expected sizes (109 bp and 343 bp) were seen in both tissues (Fig 4).

## Discussion

In order to identify new susceptibility genes for OA we studied a set of Finnish OA families using whole exome sequencing. We identified rare variants that co-segregated with the disease in two of the three studied families (Families 8 and 12). In Family 8 c.628C>G, p.Arg210Gly variant in *OLIG3* was located in the protein coding region, while in Family 12 the identified variant (c.-127G>T) was found in the 5’UTR region of the *FIP1L1* gene.

The observed p.Arg210Gly variant in *OLIG3* was estimated pathogenic by PolyPhen-2 and Mutation Taster. Arginine in this location is highly conserved in mammals and when replaced by glycine, a much smaller amino acid, the change is likely to affect the protein structure. OLIG3 is a TF that has previously been identified in association with increased rates of joint destruction in RA [26] and poor response to methotrexate treatment in early inflammatory polyarthritis [27]. The genes regulated by OLIG3 are not known, but the expression of *OLIG3* has been linked with Wnt/β-catenin signaling during neuronal development [28]. Wnt/β-catenin signaling pathway is active in the development of synovial joint [29] and has also been implicated in the pathogenesis of RA [30] and OA [31].

*FIP1L1* is a subunit of the cleavage and polyadenylation specificity factor (CPSF) protein complex, which contributes to the mRNA polyadenylation site recognition and stimulates polyadenylation. The polyadenylation of the mRNA 3’ end is an important part of the pre-mRNA processing and affects the efficiency and tissue specificity of gene expression [32,33]. Mutations interfering with 3’ end processing are known to affect human health [34]. *FIP1L1* is also associated with a fusion gene contributing to the pathogenesis of leukemia [35] and hypereosinohpilic syndrome [36].

The observed c.-127G>T variant in *FIP1L1* can affect the affinity of TFs and thus may alter the expression of *FIP1L1* in target tissues. Changes in the *FIP1L1* expression may have downstream effects on the expression of other genes via the 3’end processing. The location of variant c.-127G>T in the 5’UTR region of *FIP1L1* suggests that it may affect the binding of TFs such as IRF1, SPI1, ELF1, ELK1, GABPA and TCF3. For example, IRF1 and SPI1 have previously been linked to musculoskeletal phenotypes: The mouse homolog for IRF1 has an important role in regulating bone metabolism [37] and SPI1 influences the differentiation of precursor myeloid cells to osteoclasts [38]. In the spi1-deficient mice, differentiation of both macrophages and osteoclasts is inhibited [38].

Bone and cartilage tissues are not represented in general databases for human tissue expression such as Human Protein Atlas and Genotype-Tissue Expression portal. Therefore, we carried out a PCR analysis and observed that *FIP1L1* and *OLIG3* are indeed expressed in these tissues supporting their potential role in musculoskeletal diseases. Unfortunately we were not able to assess if the relative expression levels of these genes is different between affected and unaffected individuals as it was not possible to obtain patient samples from these families. These genes have not been reported to be differentially expressed in OA patients in earlier studies [39–42], however the variants identified in our study are rare and may require considerably large data sets to reach statistical significance in general population.

Regulatory mechanisms are known to play an important role in the pathogenesis of OA. The most well-established finding is the SNV rs143383 in the *GDF5* 5’UTR [43]. It has been shown that rs143383 affects the binding affinity of TFs leading to a repressed expression [44]. FIP1L1 and OLIG3 both participate in the regulation of transcription. Mutations in regulators of gene expression can have wide downstream effects. Even subtle regulatory changes in the gene expression can, especially over time, predispose towards pathogenic effects. Identifying the target genes and pathways in joint tissues could provide new insight into molecular background of OA and possibly help in finding new biomarkers or therapeutic targets.

The whole exome sequencing method has been shown to be capable of identifying co-segregating variants in family datasets of common diseases. In the present study the identified variants in the *OLIG3* and *FIP1L1* genes were specific to the respective families. This is not unexpected in the light of the Finnish population history of local isolates and enrichment of rare, functional alleles [16–18]. We have previously shown that specific variants associate with vertebral endplate signal changes (modic changes) in two Finnish families [45]. Functional evidence is needed, however, to understand the biological effect of the identified variants. Ideally, to further study their role would require tissue samples from patients, which were not available from the studied families. Alternatively, *in vitro* studies with general cell lines and specifically generated gene constructs could be possible. Here we utilized a bioinformatic approach to study the function and frequency of the variants. Previous studies have identified rare and low-frequency variants in association with OA [11,12] and functional evidence of the role of these genes and variants have been reported in subsequent studies [46,47].

In Family 11 we did not identify any rare, pathogenic variants co-segregating with OA. Since the whole exome sequencing data is restricted to the coding regions of the genome, it is possible or even likely that some of the predisposing variants are located elsewhere in the genome and that there are other regulatory mechanisms involved. This was actually the case in another family in the same family dataset (Family 10) where a common variant (rs11446594) in TFBS on chromosome 2q21 was identified by linkage analysis and targeted re-sequencing [19].

## Conclusions

In conclusion, we have identified two variants co-segregating with OA in Finnish families leading to the finding of novel candidate genes for OA. Based on their well-defined function in the transcription *OLIG3* and *FIP1L1* may participate in regulatory events leading to OA.

## Supporting information

S1 Table All variants that were shared by the exome sequenced family members and not found in the in-house exome set. (XLSX)
